# Supplementary material for: Oncology professionals’ perspectives towards cardiac surveillance in breast cancer patients with high cardiotoxicity risk: A qualitative study
Source: PLoS One. 2021 Mar 31;16(3):e0249067. doi: 10.1371/journal.pone.0249067 (PMC8011722; doi:10.1371/journal.pone.0249067)
Supplement: S1 Table — A table is presented with the interview guide; all main questions as well as potential follow-up questions are listed. The order of the questions is based on the seven domains of the TICD checklist, which was the theoretical basis of the interview guide. (PDF) [file pone.0249067.s001.pdf]

Interview guide healthcare professionals

Guideline factors

- Does the hospital you work in provide guidelines for cardiac surveillance of women undergoing breast cancer treatment?

  <u>If yes:</u>

- What can you tell me about the accessibility and clarity of these guidelines?

  <u>If no:</u>

- What is your knowledge of current guidelines/recommendations existing in literature for women at risk of cardiac dysfunction related to breast cancer treatment?
- What is your opinion about the feasibility of the guidelines in current practice?

Individual health professional factors

- Are you aware of the risk of cardiotoxicity in women with breast cancer related to the current treatment regimens?
- Do you evaluate the cardiovascular disease and cardiotoxicity risk for breast cancer patients prior to cancer treatment?
- Could you please elaborate why you (do not) choose to evaluate this risk?
- How often do you evaluate the cardiac function of women with breast cancer receiving cardiotoxic treatments?
- Why do you choose this particular approach?
- At what point would you choose to refer a patient to a cardiologist?
- What should cardiac surveillance for women with breast cancer encompass according to your opinion?
- In your opinion, what is necessary to achieve this?
- What are current processes that improve cardiac surveillance for this patient group?
- What are current processes that undermine optimal cardiac surveillance for this patient group?

Patient factors

- What impact, in your experience, does cardiac dysfunction have on women who are undergoing or have undergone breast cancer treatment?
- Impact regarding physical and mental health, quality of life or social and societal participation
- In your opinion, what do women with breast cancer who are at risk of developing cardiac dysfunction due to treatment need and expect regarding cardiac surveillance?
- How do patients' needs and expectations influence your clinical decision-making regarding cardiac surveillance in current practice?

Professional interaction

- How would you describe the collaboration between oncology and cardiology professionals in providing care for women with or at risk of cardiac damage due to breast cancer treatment?
- What are facilitating factors for this collaboration?
- What are barriers for this collaboration?
- To what extent do peers and managers influence you (positively or negatively) in delivering cardiac surveillance for women with breast cancer?

Incentives and resources

- What could your organization do to help motivate you in changing current cardiac surveillance for women with breast cancer?

• Are there any workplace or resource factors that facilitate or hinder you in delivering cardiac surveillance for women with breast cancer?
**Capacity for organizational change**
• How would you describe the capacity for organizational change within your hospital?<br>• How would you describe the capacity for organizational change within your team?
**Social, political and legal factors**
• Are there, in your opinion, any social, political or legal factors that influence cardiac surveillance for women with breast cancer?
