# Supplementary material for: Oncology professionals’ perspectives towards cardiac surveillance in breast cancer patients with high cardiotoxicity risk: A qualitative study
Source: PLoS One. 2021 Mar 31;16(3):e0249067. doi: 10.1371/journal.pone.0249067 (PMC8011722; doi:10.1371/journal.pone.0249067)
Supplement: Attachment — Submitted filename: Response to reviewers.docx [file pone.0249067.s002.docx]

| **Comments from Reviewer 1** | **Response** |
| --- | --- |
| Only 12 physicians were interviewed - and only 2 males. This really limits the impact of the study. At least 30 should be surveyed. | We thank the reviewer for this comment. We conducted interviews up to the point of data saturation. After the tenth interview, data saturation was reached, which means that no new codes (i.e., no codes that introduced a new topic) were added to the data. We conducted two more interviews to confirm the data saturation. After data saturation was reached, it was deemed methodologically unnecessary to conduct additional interviews because sufficient data had been collected to answer the specific research question.  We have revised the methods and results sections to clarify this concern.  Methods (P 6, Ln 11-18):  *Data saturation was defined as the point where additional interviews did not lead to any codes that introduced a new topic, which was reached after ten interviews. Two more interviews confirmed saturation and ensured maximum variation in the sample.*  *After transcribing the interviews, 740 codes were generated and grouped in 32 clusters, such as multidisciplinary collaboration, continuity of care, burden, need for knowledge, urgency, logistics, awareness, personalized care, research, visibility of late cardiac effects, and cost effectiveness. The recurring themes within these clusters were discussed to identify the final themes to answer the research question.*  The limited variation in gender of the interviewees is a reflection of the gender distribution of breast cancer professionals in general. Most oncology professionals with a specific focus on breast cancer patients are female.  To clarify these considerations, we revised the methods section (P 4, Ln 12-13):  *A purposive sample with maximum variation in gender, work experience, and hospital type was selected with the aim to reflect the true variation in characteristics observed in clinical practice.* |
| The manuscript should be edited by someone who is a native speaker of English. | The manuscript had been checked by a native speaker before submission to PLOS ONE. Following reviewers’ comments, we asked a second native speaker to edit the manuscript, and any remaining grammatical errors were corrected. |

| **Comments from Reviewer 2** | **Response** |
| --- | --- |
| Only comment is regarding the monodisciplinary approach. The manuscript can be strengthened by expanding the group of interviews to cardiologists/specialists in vascular medicine and radiation oncologists. | We thank the reviewer for this comment, we agree that professionals from cardiology, vascular medicine, and radiotherapy departments could provide additional perspectives on cardiac surveillance.  However, with this study, we primarily aimed to explore the perceptions of oncology professionals because they are often the “gatekeepers” for the oncological care trajectories in the Netherlands.  Oncologists or oncology nurse practitioners are the first professionals with whom a patient has contact once they are diagnosed with a malignancy. They provide a first draft of a patient’s treatment plan after which the patient is referred to a radiotherapist and surgeon, if indicated.  Subsequently, with multidisciplinary discussion and further diagnostic procedures, the treatment plan is finalized. We believe that this would also be an ideal moment for oncologists to assess whether a patient has an increased cardiovascular risk based on their baseline characteristics and the scheduled treatments, and – if indicated – refer the patient to a cardiologist.  Additionally, a previous study suggests that, in current practice, patients rarely receive any form of cardiac surveillance. Therefore, we were primarily interested in the perceptions of oncology professionals and potential influencing factors of delivering cardiac surveillance. |
| It is not clear why an epidemiologist was included in an interview about clinical practices. | The epidemiologist included in our study is employed at a hospital specialized in oncological care; her work primarily focuses on improving breast cancer care and delivering cardiac surveillance in practice. This participant closely works with oncologists to improve current practice and has a wide knowledge of cardiac surveillance and its influencing factors. Therefore, she was deemed eligible for study participation. |
